# Real-World Use, Prescribing Patterns, and Short-Term Clinical Evolution of Extensively Hydrolyzed and Hydrolyzed Rice Formulas in Infants with Cow’s Milk Protein Allergy: An Analysis from the ETAPA Project

**DOI:** 10.3390/nu18132137

**Published:** 2026-07-02

**Authors:** Juan José Díaz-Martín, Rafael Martín-Masot, Alicia Santamaría-Orleans, Víctor Manuel Navas-López

**Affiliations:** 1 Unit of Pediatric Gastroenterology and Nutrition, Hospital Universitario Central de Asturias, 33011 Oviedo, Spain; diazmjuan@uniovi.es; 2 Pediatric Gastroenterology and Nutrition Unit, Hospital Regional Universitario de Málaga, 29010 Málaga, Spain; victorm.navas.sspa@juntadeandalucia.es; 3 Medical Department, Laboratorios Ordesa S.L., 08038 Barcelona, Spain

**Keywords:** cow’s milk protein allergy, hydrolyzed rice formula, extensively hydrolyzed formula, CoMiSS, infants, real-world evidence, prescribing patterns, nutritional management

## Abstract

**Background:** Hydrolyzed rice formulas (HRFs) are increasingly recognized as an alternative nutritional option for infants with cow’s milk protein allergy (CMPA), but real-world data on prescribing patterns and early clinical evolution remain limited. **Objective**: This study aimed to describe real-world use and prescribing patterns of HRF and eHF in infants with CMPA, identify factors associated with HRF recommendation, and assess short-term clinical evolution after formula use. **Methods:** This observational analysis from the ETAPA project included two mutually exclusive infant-level cohorts: a prospective recommendation cohort assessing variables associated with HRF versus eHF recommendation, and a retrospective treated cohort assessing 7-day clinical evolution after formula use. In the retrospective cohort, the main clinical outcome was absolute change in CoMiSS from day 0 to day 7. A propensity score-weighted sensitivity analysis was performed to address measured confounding by indication. **Results:** Overall, 1505 valid infant-level records were analyzed: 1094 in the prospective cohort and 411 in the retrospective cohort. In the prospective cohort, HRF was recommended in 214/1094 records (19.6%). In multi-variable analysis, older infant age was associated with higher odds of HRF recommendation (OR 1.034 per month, 95% CI 1.002–1.067; *p* = 0.036), whereas IgE-mediated CMPA was associated with lower odds (OR 0.673, 95% CI 0.459–0.988; *p* = 0.043). In the retrospective cohort, CoMiSS decreased markedly in both groups (eHF: median reduction 7.0 [IQR 4.0–10.0] points; HRF: 6.5 [4.0–10.0] points; *p* = 0.661). After adjustment, HRF was not associated with a statistically significant difference in absolute CoMiSS reduction compared with eHF (β −0.513 points, 95% CI −1.108 to 0.082; *p* = 0.091). **Conclusions:** In routine pediatric practice, HRF was used across a broad range of CMPA profiles and was associated with clinically relevant short-term symptom improvement. These findings support HRF as an additional nutritional option for CMPA management, while the observational design precludes conclusions of equivalence or non-inferiority versus eHF.

## 1. Introduction

Cow’s milk protein allergy (CMPA) is one of the most frequent food allergies during infancy and early childhood, with an estimated incidence of approximately 2–3% during the first year of life in developed countries, although prevalence estimates vary according to diagnostic criteria, population, and confirmation strategy [1,2,3]. From a clinical and pathophysiological perspective, CMPA encompasses heterogeneous phenotypes, broadly classified into IgE-mediated forms, usually characterized by immediate cutaneous, respiratory, or systemic manifestations, and non-IgE-mediated forms, which are more commonly associated with delayed gastrointestinal symptoms [1,2]. This heterogeneity, together with the substantial overlap between CMPA-related symptoms and common functional gastrointestinal disorders of infancy, contributes to both overdiagnosis and underdiagnosis in routine practice [1,2,4].

Current international guidance emphasizes that CMPA diagnosis should not rely solely on symptoms or sensitization tests. Instead, diagnosis generally requires clinical improvement after a diagnostic elimination diet followed by reintroduction or oral food challenge, except in selected high-risk situations such as severe reactions or strongly suggestive IgE-mediated disease [2,4]. The WAO DRACMA update recommends a diagnostic elimination period of approximately 1–2 weeks for IgE-mediated CMPA and 2–4 weeks for non-IgE-mediated CMPA before reintroduction is considered [4]. However, implementation of these recommendations remains variable in real-world settings, particularly across primary care and hospital-based pediatric practice [5,6,7].

The Cow’s Milk-related Symptom Score (CoMiSS) was developed as an awareness tool to help healthcare professionals recognize symptoms potentially attributable to cow’s milk in infants. It includes crying, regurgitation, stool consistency, skin manifestations, and respiratory symptoms, with a total score ranging from 0 to 33 [8,9,10]. Importantly, CoMiSS should not be regarded as a stand-alone diagnostic test for CMPA. Prospective validation studies, including the multicenter MOSAIC trial, did not demonstrate sufficient accuracy to support its isolated diagnostic use [11,12]. In presumed healthy infants aged up to 6 months, the median CoMiSS is approximately three, and the 95th percentile is nine, which contributed to the 2022 expert recommendation to lower the proposed positive cut-off from ≥12 to ≥10 [10,13]. Nevertheless, validation studies have reported variable diagnostic performance across populations, and lower optimal thresholds have been suggested in some cohorts [14]. Therefore, CoMiSS may be useful for structured symptom assessment and monitoring clinical evolution after dietary intervention, if it is interpreted within the broader clinical context.

Nutritional management is central to CMPA care in non-breastfed infants. Extensively hydrolyzed cow’s milk formulas (eHF) have traditionally been used as the standard first-line option, whereas amino acid formulas (AAF) are generally reserved for severe, complex, or refractory cases [1,2]. In recent years, hydrolyzed rice formulas (HRF) have gained increasing attention as an alternative option for the dietary management of CMPA. Current WAO DRACMA and ESPGHAN recommendations suggest either eHF or HRF as first-option choices for non-breastfed infants with IgE- or non-IgE-mediated CMPA, followed by AAF as a second option and soy formula as a third option; however, these recommendations are conditional because the certainty of comparative evidence remains very low [1,2,15,16].

The available evidence on HRFs is encouraging but still limited. Early clinical studies and more recent comparative data have reported adequate growth, acceptable safety, good tolerance, and symptom improvement in infants with CMPA receiving HRFs [17,18,19,20]. Nevertheless, direct comparative evidence between HRFs and eHFs remains sparse, and existing studies do not allow firm conclusions regarding equivalence or non-inferiority [16,20]. In addition, the literature on real-world formula selection is largely consensus-based, with limited quantitative evidence on how patient phenotype, symptom burden, prescriber characteristics, and healthcare setting influence formula choice [21,22].

Therefore, this analysis from the ETAPA project aimed to: (i) describe real-world recommendations and use of eHF and HRF in infants with CMPA; (ii) explore infant-, disease-, and prescriber-related variables associated with HRF recommendation versus eHF; and (iii) assess short-term clinical evolution over 7 days among infants treated with either formula.

## 2. Materials and Methods

### 2.1. Study Design

The ETAPA project (Survey on Tolerance Acquisition in cow’s milk protein allergy in Primary and Hospital Pediatric Care) was an observational real-world project conducted among pediatricians working in primary care and hospital-based pediatric units across Spain, including both public and private healthcare settings [6]. The original project included a structured survey of pediatric practice, and two infant-level registry components designed to describe formula recommendation and clinical evolution in infants with CMPA.

For the present analysis, two complementary cohorts were evaluated. The prospective recommendation cohort included infants for whom participating pediatricians recorded a formula recommendation for CMPA management. This cohort was analyzed to characterize prescribing patterns and factors associated with recommending HRF rather than eHF. The retrospective treated cohort included infants who had already received one of the study formulas and for whom short-term clinical evolution from day 0 to day 7 was recorded. This cohort was analyzed to evaluate early clinical evolution and tolerability according to the formula received. The prospective recommendation cohort and the retrospective treated cohort were mutually exclusive; each infant-level record contributed to only one analytical cohort.

### 2.2. Participants and Data Collection

Pediatricians were invited to participate through a national pediatric database maintained by Laboratorios Ordesa S.L. (Barcelona, Spain), as described in the previous ETAPA publication [6]. Candidates regularly involved in the diagnosis and management of CMPA and who provided consent to participate were included. Participation was voluntary and anonymous and required prior completion of a registration form in which participants explicitly agreed to take part in the study and consented to participate.

Because this was a pragmatic real-world registry, the study did not mandate oral food challenge, standardized elimination–reintroduction procedures, or centralized diagnostic adjudication. Therefore, CMPA status reflected the participating pediatrician’s routine clinical diagnosis, and the registry did not systematically capture mutually exclusive categories such as challenge-confirmed CMPA, diagnosis after elimination diet alone, or diagnosis based solely on clinical judgment.

### 2.3. Variables and Outcomes

Prescriber-level variables included age, sex, work setting (public, private, or both), type of healthcare facility, and specialty. Pediatric gastroenterologists and pediatric allergologists were grouped as gastroenterology/allergology specialists (G/PA) for selected analyses. Infant-level variables included sex, age, age at diagnosis, CMPA phenotype, physician-reported clinical severity, baseline CoMiSS, and formula type. CMPA phenotype was recorded by the participating pediatrician as IgE-mediated or non-IgE-mediated according to routine clinical assessment. IgE-mediated CMPA generally referred to cases with immediate symptoms and/or evidence of IgE sensitization when available, whereas non-IgE-mediated CMPA generally referred to delayed clinical presentations, predominantly gastrointestinal, without evidence of an IgE-mediated mechanism. No centralized adjudication or study-mandated confirmatory testing was performed. Disease severity was recorded as mild, moderate, severe, or very severe according to the participating physician’s global clinical judgment at the time of data entry. This variable should therefore be interpreted as physician-reported clinical severity rather than as a classification based on a prespecified validated severity scale. For multivariable and propensity score models, this categorical variable was coded as an ordinal score (1 = mild, 2 = moderate, 3 = severe, 4 = very severe) and is reported in tables and models as the physician-reported ordinal severity score; both terms refer to the same physician-reported severity variable. CoMiSS and individual domain scores were recorded by the participating pediatrician using the standard CoMiSS domains [10]. Scores were based on information available in routine clinical practice, including physician assessment, caregiver report, and/or medical records, as applicable. No centralized reassessment of CoMiSS domains was performed.

The formulas were analyzed as eHF or HRF according to the product recorded in the database. Because commercially available HRFs and eHFs formulas may differ in composition, this analysis focused on two formulas produced by the same manufacturer, Laboratorios Ordesa S.L. The main intended difference between the two products was the protein fraction, whereas the remaining ingredients and overall nutritional profile were broadly comparable. A summary of the main nutritional characteristics of the two formulas, including energy density, protein source and content, fat composition, carbohydrate composition, and selected micronutrients, is provided in Supplementary Table S1.

Commercial brand names were not used in the manuscript. In the prospective cohort, formula type represented the pediatrician’s recommendation. In the retrospective cohort, formula type represented the formula already used by the infant. CoMiSS was used as a structured symptom score to quantify baseline symptom burden and short-term clinical evolution. In this study, CoMiSS was not used as a stand-alone diagnostic criterion for CMPA, but as a standardized measure of symptom burden and response to dietary management. Baseline CoMiSS was analyzed both as a continuous variable and as a categorical variable using the ≥12 threshold, in line with the earlier literature, and to allow comparison with previous studies.

In the retrospective cohort, clinical outcomes included CoMiSS at day 0 and day 7, absolute and percentage CoMiSS reduction, reduction of at least 50% in CoMiSS, CoMiSS <6 at day 7, persistence of CoMiSS ≥12 at day 7, change in CoMiSS domains, days until observed improvement, global clinical evolution assessed on a 1–4 scale, and product satisfaction assessed on a 1–4 scale. On day 7, CoMiSS and symptom-domain variables were not interpreted in the prospective cohort, as they were not measured as follow-up outcomes in that component. The 7-day time point was selected to capture early symptom evolution in routine practice and should be interpreted as a pragmatic early-response endpoint rather than as an assessment of sustained effectiveness, nutritional safety, growth, or tolerance acquisition.

### 2.4. Statistical Analysis

Categorical variables are summarized as absolute frequencies and percentages. Continuous variables were assessed for normality using visual inspection and the Shapiro–Wilk test. Normally distributed variables are presented as mean and standard deviation (SD), whereas non-normally distributed variables are presented as median and interquartile range (IQR). Between-group comparisons were performed using Student’s t-test or the Mann–Whitney U test, as appropriate. All tests were two-sided, and *p* < 0.05 was considered statistically significant.

Multicollinearity was assessed using variance inflation factors (VIFs), with values >5 considered indicative of potentially relevant collinearity. Model assumptions were evaluated using residual diagnostics for linear models and standard goodness-of-fit and calibration measures for logistic models. The distribution of propensity scores and stabilized inverse probability of treatment weights was inspected to assess covariate overlap and identify potential extreme weights.

Phenotype-stratified analyses were considered exploratory. Formal interaction testing was not performed because the study was not powered for interaction analyses, and subgroup sample sizes were limited.

**Figure 1:**
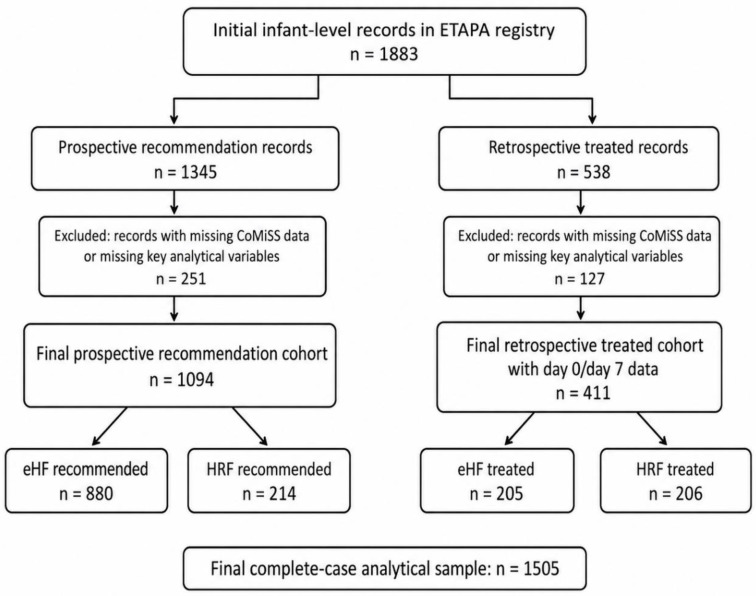
Flow diagram of infant-level records included in the ETAPA analysis. The initial registry included 1883 infant-level records, comprising 1345 prospective recommendation records and 538 retrospective treated records. Records with missing CoMiSS data or missing key analytical variables were excluded, resulting in a final complete-case analytical sample of 1505 records: 1094 in the prospective recommendation cohort, including 880 eHF recommendations and 214 HRF recommendations, and 411 in the retrospective treated cohort with day 0/day 7 data, including 205 infants treated with eHF and 206 treated with HRF. CoMiSS, Cow’s Milk-related Symptom Score; eHF, extensively hydrolyzed formula; HRF, hydrolyzed rice formula.

All adjusted models were conducted using complete cases for the variables included in each model. The number of excluded records by cohort is shown in Figure 1. Because exclusion was mainly driven by missing CoMiSS data or missing key analytical variables, complete-case analyses were considered appropriate for the prespecified analytical objectives, although the potential for selection bias was acknowledged.

To further address potential confounding by indication in the retrospective treated cohort, a propensity score-weighted sensitivity analysis was performed. This analysis was restricted to the retrospective treated cohort because this was the only cohort evaluating clinical outcomes after formula exposure. In the prospective recommendation cohort, formula recommendation was the outcome of interest; therefore, propensity score adjustment was not conceptually applicable.

The propensity score was estimated using logistic regression with formula received (HRF versus eHF) as the dependent variable and prespecified baseline covariates, including infant age, infant sex, age at diagnosis, CMPA phenotype, physician-reported ordinal severity score, baseline CoMiSS, prescriber age, G/PA specialist profile, work setting, and type of facility. Stabilized inverse probability of treatment weights were then calculated. Covariate balance before and after weighting was assessed using standardized mean differences, with values < 0.10 considered indicative of adequate balance. The association between formula type and absolute CoMiSS reduction from day 0 to day 7 was re-estimated using propensity score-weighted regression models with robust standard errors. Secondary outcomes were analyzed analogously. Because propensity score methods can only account for measured covariates, residual confounding by unmeasured factors cannot be excluded.

In the prospective recommendation cohort, multivariable logistic regression was used to identify factors independently associated with HRF recommendation versus eHF. Candidate covariates included infant age, CMPA phenotype, physician-reported ordinal severity score, baseline CoMiSS ≥12, prescriber age, G/PA specialist profile, work setting, and type of facility. In the retrospective cohort, adjusted linear regression was used to assess the association between formula type and absolute CoMiSS reduction from day 0 to day 7. Logistic regression was used for CoMiSS reduction ≥ 50%. Analyses were performed using complete cases for each model.

All analyses were carried out using IBM SPSS Statistics version 27.0 for MAC (IBM Corp., Armonk, NY, USA).

## 3. Results

### 3.1. General Characteristics of Participants

A total of 269 pediatricians participated in the study (mean age 48.3 ± 11.0 years; 62.3% female). Most respondents worked in the public sector (60.1%), 19.8% in private practice, and 20.2% in both. Urban environments accounted for the majority of work settings (87.8%). Workplaces included primary care centers (55.4%), public hospitals (20.1%), and private clinics (17.8%). Most participants specialized in general pediatrics (71.4%), with smaller groups in pediatric gastroenterology (19.1%) and allergology (4.2%) (Supplementary Table S2).

### 3.2. Study Population

From an initial registry of 1883 infant-level records, including 1345 prospective recommendation records and 538 retrospective treated records, records with missing CoMiSS data or missing key analytical variables were excluded. The final complete-case analytical sample comprised 1505 infant-level records, including 1094 records in the prospective recommendation cohort and 411 records in the retrospective treated cohort. The prospective recommendation cohort comprised 1094 records, of which 880 (80.4%) corresponded to eHF recommendation and 214 (19.6%) to HRF recommendation. The retrospective treated cohort comprised 411 records with day 0 and day 7 clinical evolution data, including 205 infants treated with eHF and 206 treated with HRF (Figure 1). This complete-case approach was considered appropriate for a real-world observational analysis, in which the availability of specific variables may vary according to routine clinical documentation and the analytical objective.

Overall, median infant age was 5.0 [IQR 3.0–8.0] months and median age at diagnosis was 2.0 [1.0–4.0] months. Most records corresponded to non-IgE-mediated CMPA (70.4%), and most cases were classified as mild or moderate. Baseline CoMiSS was 10.0 [7.0–13.0], and 35.0% of infants had baseline CoMiSS ≥12. Infant characteristics by cohort are summarized in Table 1.

### 3.3. Prospective Recommendation Cohort: Factors Associated with HRF Recommendation

In the prospective recommendation cohort, infants for whom HRF was recommended were slightly older than those for whom eHF was recommended (6.0 [3.0–8.5] vs. 5.0 [3.0–7.0] months; *p* = 0.010), with an older age-at-diagnosis distribution (3.0 [2.0–5.0] vs. 2.0 [1.0–4.0] months; *p* = 0.034). Among HRF recommendations, 75.2% corresponded to non-IgE-mediated and 24.8% to IgE-mediated CMPA, compared with 68.8% and 31.2%, respectively, among eHF recommendations (*p* = 0.084). Baseline CoMiSS, the proportion of infants with CoMiSS ≥12, severity distribution, prescriber specialty, work setting, and facility type were not significantly different between recommended formula groups (Table 2).

**Figure 2:**
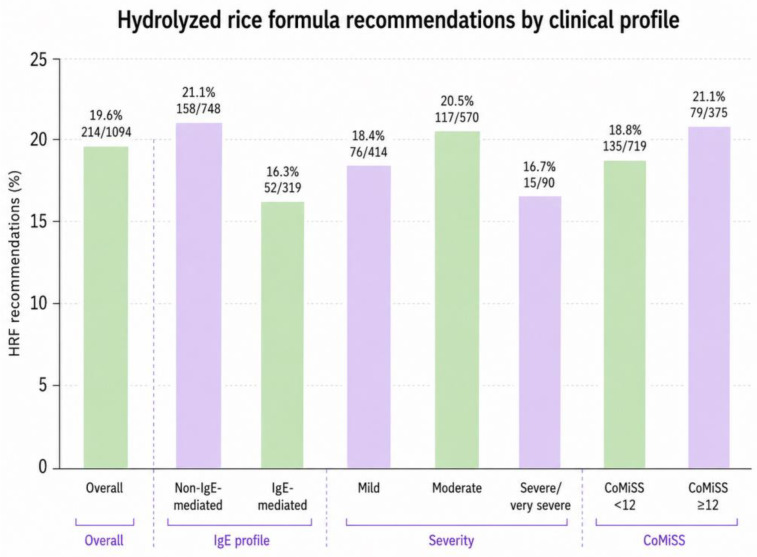
Percentage of hydrolyzed rice formula recommendations in the prospective cohort according to clinical profile. CoMiSS, Cow’s Milk-related Symptom Score; HRF, hydrolyzed rice formula; IgE, immunoglobulin E.

The proportion of HRF recommendations was 19.6% overall, 21.1% in non-IgE-mediated CMPA, and 16.3% in IgE-mediated CMPA. HRF recommendations were observed across all severity categories and across both CoMiSS categories (Figure 2). In the adjusted logistic regression model, older infant age was independently associated with higher odds of HRF recommendation (OR 1.034 per month, 95% CI 1.002–1.067; *p* = 0.036), whereas IgE-mediated CMPA was associated with lower odds of HRF recommendation compared with non-IgE-mediated CMPA (OR 0.673, 95% CI 0.459–0.988; *p* = 0.043). Baseline CoMiSS ≥12, clinical severity, prescriber age, G/PA specialist profile, work setting, and type of facility were not significantly associated with HRF recommendation (Table 3).

### 3.4. Retrospective Treated Cohort: Baseline Comparability

The retrospective treated cohort included 205 infants treated with eHF and 206 treated with HRF. Baseline age, CMPA phenotype, severity score, prescriber characteristics, and healthcare setting were broadly comparable between groups. Infants in the HRF group had a slightly older age-at-diagnosis distribution than those in the eHF group (2.0 [1.0–4.0] vs. 2.0 [1.0–3.0] months; *p* = 0.022) and a higher proportion of female infants. Baseline CoMiSS was similar between groups (10.0 [7.0–13.0] vs. 10.0 [7.0–13.0]; *p* = 0.127). Baseline characteristics are shown in Table 4. These baseline differences, particularly sex distribution, age at diagnosis, and the higher proportion of infants with baseline CoMiSS ≥12 in the HRF group, were considered clinically relevant potential sources of confounding and were therefore accounted for in adjusted and sensitivity analyses when applicable.

### 3.5. Seven-Day Clinical Evolution in the Retrospective Cohort

Both formula groups showed marked symptom improvement from day 0 to day 7. Median CoMiSS decreased from 10.0 [7.0–13.0] to 2.0 [1.0–4.0] in the eHF group and from 10.0 [7.0–13.0] to 4.0 [1.0–5.8] in the HRF group. The absolute CoMiSS reduction was similar between groups (7.0 [4.0–10.0] with eHF vs. 6.5 [4.0–10.0] with HRF; *p* = 0.661). Percentage CoMiSS reduction was higher in the eHF group (76.5% [57.1–90.0] vs. 69.2% [50.0–83.3]; *p* = 0.003), and the proportion of infants with CoMiSS <6 at day 7 was also higher with eHF (83.9% vs. 74.8%; *p* = 0.028). However, persistence of CoMiSS ≥12 at day 7 was uncommon in both groups (2.0% vs. 1.5%; *p* = 0.724).

**Figure 3:**
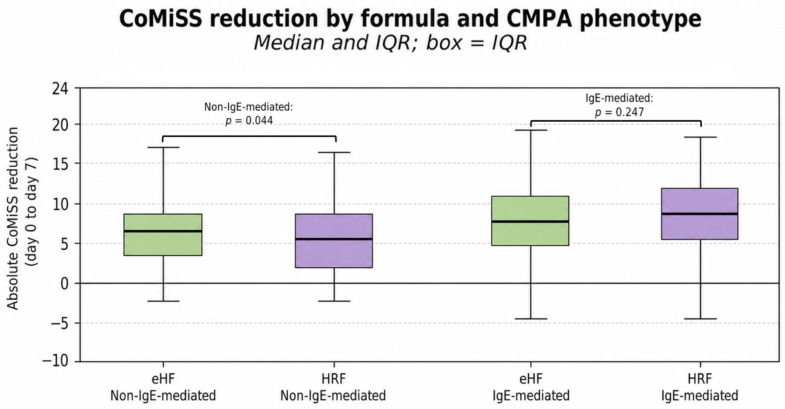
Absolute CoMiSS reduction from day 0 to day 7 according to the formula and CMPA phenotype. Boxes represent interquartile ranges; horizontal lines represent medians; whiskers represent the observed range. CMPA, cow’s milk protein allergy; CoMiSS, Cow’s Milk-related Symptom Score; eHF, extensively hydrolyzed formula; HRF, hydrolyzed rice formula; IQR, interquartile range.

Across CoMiSS domains, both groups improved in crying, regurgitation, stool score, eczema, urticaria, and respiratory symptoms. The regurgitation domain showed a statistically significant between-group difference in rank distribution (*p* = 0.003) despite identical median reductions, whereas changes in the remaining domains were broadly similar. In subgroup analyses by CMPA phenotype, CoMiSS reduction was observed in both formula groups and in both IgE-mediated and non-IgE-mediated CMPA (Table 5 and Table 6, and Figure 3).

### 3.6. Days to Improvement, Global Evolution, and Satisfaction

**Figure 4:**
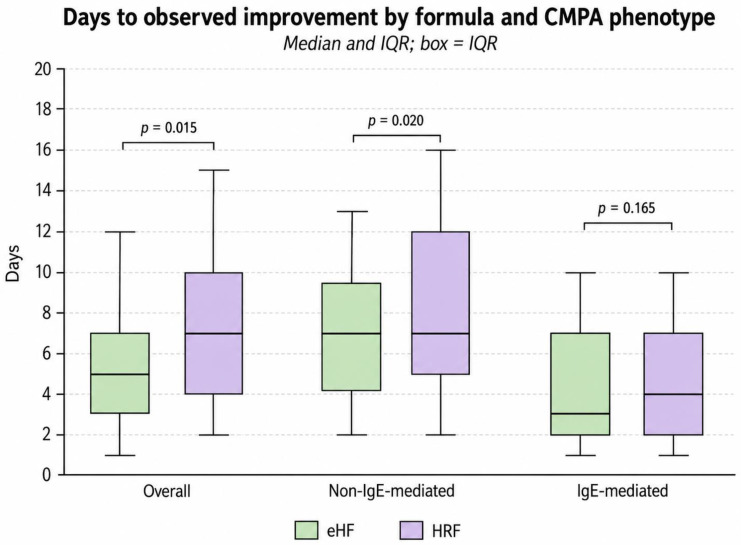
Days to observe clinical improvement according to the formula and CMPA phenotype. Boxes represent interquartile ranges; horizontal lines represent medians; whiskers represent the observed range. CMPA, cow’s milk protein allergy; eHF, extensively hydrolyzed formula; HRF, hydrolyzed rice formula; IQR, interquartile range.

The median number of days until observed clinical improvement was 5.0 [3.0–7.0] in the eHF group and 7.0 [4.0–10.0] in the HRF group (*p* = 0.015). Global clinical evolution and product satisfaction were high in both groups and did not differ significantly between formulas. Median global evolution scores were 3.0 [3.0–4.0] for eHF and 3.0 [3.0–4.0] for HRF (*p* = 0.318), while satisfaction scores were 4.0 [3.0–4.0] and 4.0 [3.0–4.0], respectively (*p* = 0.158). Similar patterns were observed in phenotype-stratified analyses (Table 6 and Figure 4).

### 3.7. Adjusted Clinical Outcome Models in the Retrospective Cohort

In the adjusted linear regression model for absolute CoMiSS reduction, HRF was not significantly associated with a different reduction compared with eHF (β −0.513 points, 95% CI −1.108 to 0.082; *p* = 0.091). Baseline CoMiSS was strongly associated with greater absolute reduction (β 0.786 points per baseline point, 95% CI 0.705–0.866; *p* < 0.001). In the adjusted logistic regression model, HRF was associated with lower odds of achieving CoMiSS reduction ≥ 50% (OR 0.576, 95% CI 0.335–0.989; *p* = 0.045), although this exploratory finding should be interpreted cautiously because this analysis was observational and may be affected by baseline differences and residual confounding.

To facilitate interpretation of the main comparative findings across the two analytical cohorts, Supplementary Table S3 summarizes the principal differences observed between eHF and HRF in terms of recommendation patterns, baseline characteristics, short-term clinical evolution, and adjusted outcomes.

### 3.8. Propensity Score-Weighted Sensitivity Analysis

In the propensity score-weighted sensitivity analysis, 357 complete cases from the retrospective treated cohort were included, comprising 179 infants treated with eHF and 178 treated with HRF. Propensity scores ranged from 0.25 to 0.80, and stabilized inverse probability of treatment weights ranged from 0.62 to 2.43, suggesting adequate covariate overlap and no extreme weights.

Before weighting, the largest imbalances were observed for infant sex, age at diagnosis, baseline CoMiSS, and CMPA phenotype. After stabilized inverse probability weighting, covariate balance improved substantially, with all standardized mean differences below 0.03 (Supplementary Table S4). No relevant multicollinearity was detected in the adjusted models or in the propensity score model, with all VIF values below 5.

The propensity score-weighted estimate for the association between HRF and absolute CoMiSS reduction was directionally consistent with the primary adjusted model and did not show a statistically significant difference compared with eHF (β −0.49 points, 95% CI −1.52 to 0.54; *p* = 0.353). HRF was also not significantly associated with day-7 CoMiSS (β 0.52 points, 95% CI −0.14 to 1.19; *p* = 0.123), CoMiSS day 7 <6 (OR 0.68, 95% CI 0.41 to 1.13; *p* = 0.139), days to observed improvement (β 1.30 days, 95% CI −0.46 to 3.06; *p* = 0.149), or global clinical evolution (β −0.07, 95% CI −0.22 to 0.07; *p* = 0.318). However, HRF was associated with lower odds of achieving a CoMiSS reduction ≥ 50% (OR 0.56, 95% CI 0.33 to 0.95; *p* = 0.032), and percentage CoMiSS reduction showed a non-significant trend favoring eHF (β −6.94%, 95% CI −14.53 to 0.65; *p* = 0.073). These findings support the robustness of the primary analysis regarding absolute CoMiSS reduction, while reinforcing the need for cautious interpretation of secondary comparative outcomes.

## 4. Discussion

In this real-world analysis from the ETAPA project, HRF was recommended and used across a broad range of infants with CMPA, particularly in slightly older infants and less frequently in IgE-mediated CMPA. In the retrospective treated cohort, both HRF and eHF were associated with marked short-term improvement in CoMiSS and related symptom domains after 7 days. Although the absolute CoMiSS reduction was broadly comparable between groups, some secondary outcomes favored eHF, including a greater percentage CoMiSS reduction (76.5 [57.1–90.0]% vs. 69.2 [50.0–83.3]%) and a shorter median time to observed clinical improvement (5.0 [3.0–7.0] vs. 7.0 [4.0–10.0] days). However, these findings should be interpreted cautiously because treatment allocation was not randomized, residual confounding and confounding by indication cannot be excluded, and the study was not designed to demonstrate superiority, non-inferiority, or equivalence. Therefore, the present findings should be interpreted as real-world effectiveness and prescribing-pattern data rather than definitive comparative efficacy evidence.

The prospective cohort provides a pragmatic view of formula selection in routine practice. Older infant age was independently associated with higher odds of HRF recommendation, whereas IgE-mediated CMPA was associated with lower odds. In contrast, baseline CoMiSS ≥12, physician-reported clinical severity, prescriber specialty, work setting, and facility type were not significantly associated with formula recommendation. These findings suggest that HRF is not restricted to infants with lower symptom burden or milder disease, although clinicians may remain more cautious when managing IgE-mediated CMPA. The association between older infant age and HRF recommendation may reflect practical considerations related to formula acceptance. HRF is generally perceived as having better palatability than eHF, and prescribers may anticipate greater rejection of eHF in older infants, in whom taste acceptance can become more relevant. However, because palatability, previous formula refusal, caregiver preference, and acceptance were not directly measured, this interpretation remains speculative and should be regarded as hypothesis-generating. Similarly, the lower likelihood of HRF recommendation in IgE-mediated CMPA may reflect greater perceived acute allergic risk, leading prescribers to favor more established eHF strategies.

The available literature on HRF is reassuring but remains relatively small. In an early randomized pilot study, D’Auria et al. evaluated 16 infants with CMPA aged 6–14 months who received either HRF or soy formula for 6 months; both groups showed normal growth, no adverse reactions, and normal biochemical parameters, suggesting that rice-hydrolysate formula may be nutritionally suitable in this population [17]. Subsequent prospective studies by Vandenplas et al. also reported reassuring results. In a one-month prospective study including 39 infants with confirmed CMPA, all infants tolerated the extensively hydrolyzed rice protein formula and maintained normal growth [18]. In another prospective cohort of 40 infants with challenge-confirmed CMPA followed for 6 months, the formula was tolerated by more than 90% of infants, symptom scores decreased from the first month, and weight-related z-scores normalized during follow-up [19].

More recently, the GRITO randomized comparative trial provided the most relevant direct evidence comparing HRF with eHF. In that study, 105 children with CMPA were followed for 12 months, and HRF was associated with appropriate growth, a good safety profile, and no significant between-group differences compared with eHF [20]. However, the trial should not be interpreted as proving equivalence or non-inferiority, as this was not its primary statistical design. This distinction is essential when interpreting our own findings: the absence of statistically significant differences in several outcomes is reassuring but does not establish that the two formula strategies are therapeutically equivalent.

Recent guidelines and consensus documents increasingly recognize HRF as a clinically relevant option for the dietary management of CMPA, although their recommendations remain appropriately cautious. The ESPGHAN position paper on the diagnosis, management, and prevention of CMA states that cow ’s-milk-derived eHF remains the first-choice formula for therapeutic elimination diets in formula-fed infants, while HRF can be considered as an alternative to cow-milk-derived eHF, acknowledging that randomized evidence with HRF remains less extensive [2]. In line with this, the WAO consensus on HRF stated that these formulas have demonstrated hypoallergenicity and may be recommended as a first-line option in settings where they are available [23]. Similarly, the MENAP consensus supported HRF as a first-line alternative to cow ’s-milk-based eHF in appropriate clinical contexts [24]. However, the 2024 WAO DRACMA systematic review and guideline update emphasized that comparative evidence across formula types remains of very low certainty, even though no clear differences were identified between eHF and HRF [1,15,16]. Taken together, these guidelines and consensus positions align well with the present real-world findings, which support HRF as an additional clinically useful option rather than as a proven superior, equivalent, or non-inferior alternative to eHF.

A distinctive contribution of ETAPA is the analysis of prescribing behavior. The previous literature on formula selection is largely based on consensus, expert opinion, and implementation frameworks rather than quantitative observational models. Decision-oriented publications have emphasized that formula choice may be influenced by cost, palatability, nutritional adequacy, local availability, caregiver preferences, cultural or religious considerations, persistent symptoms despite prior interventions, faltering growth, and taste acceptance [21,22]. In contrast, few studies have empirically assessed which patient- or prescriber-related factors are associated with the choice of HRF versus eHF. Our findings, therefore, add quantitative real-world evidence to an area in which recommendations necessarily allow room for individualized decision-making. The imbalance between eHF and HRF recommendations in the prospective cohort is also clinically informative. Approximately four out of five recommendations corresponded to eHF, whereas HRF accounted for around one out of five recommendations. This distribution likely reflects routine practice in Spain, where cow-milk-based extensively hydrolyzed formulas remain the most frequently used option, while hydrolyzed rice formulas represent an increasingly used alternative. Therefore, the observed distribution should not be interpreted as a limitation alone, but also as a real-world signal of current prescribing patterns.

Confounding by indication remains a central limitation when interpreting the comparative outcomes of the retrospective cohort. Although multivariable adjustment and propensity score-weighted sensitivity analyses were performed, formula choice was not randomized and may have been influenced by clinical and non-clinical factors that were not captured in the registry. These may include prior formula refusal, palatability, caregiver preference, socioeconomic factors, formula availability, previous response to dietary interventions, and the clinician’s perception of allergic risk.

The propensity score-weighted analysis substantially improved balance across measured baseline covariates and supported the primary finding that HRF was not associated with a statistically significant difference in absolute CoMiSS reduction compared with eHF. However, some secondary outcomes continued to show numerically or statistically more favorable early evolution in the eHF group. Therefore, the comparative findings should be interpreted as adjusted real-world associations rather than as estimates of causal treatment effects.

The use of CoMiSS in this study deserves specific consideration. CoMiSS was used to monitor short-term clinical evolution after formula initiation, consistent with its role as an awareness and symptom-monitoring tool rather than as a diagnostic instrument [8,10]. Several studies support its usefulness for monitoring symptom change after cow’s milk elimination, with significant score reductions reported after dietary intervention [25,26]. The magnitude of CoMiSS reduction observed in the ETAPA retrospective cohort is directionally consistent with previous real-world studies. For example, in a four-country European cohort, mean CoMiSS decreased from 11 at baseline to 4 after approximately three weeks of dietary intervention [26]. ETAPA differs from most prior studies because symptom evolution was assessed at 7 days, whereas most intervention-monitoring studies evaluate response after 2–4 weeks or at one month. Thus, the present analysis provides pragmatic early-response data, while the short observation period should also be considered a limitation.

The formula-specific CoMiSS analysis is also relevant. The literature search did not identify previous studies specifically evaluating early CoMiSS trajectories in HRF versus eHF groups. ETAPA therefore adds novel exploratory information on early symptom evolution according to formula choice in routine pediatric practice. The similar absolute reduction observed between formula groups supports the clinical usefulness of HRF in the short term, but adjusted and secondary outcomes highlight the need for cautious interpretation and further comparative research.

### Strengths and Limitations

The main strengths of this study include the large real-world sample, the integration of two complementary cohorts, and the ability to analyze both prescriber behavior and early clinical evolution. The prospective recommendation cohort provides information on how pediatricians choose formulas in routine care, while the retrospective treated cohort provides pragmatic symptom-evolution data at 7 days. In addition, the analysis included prescriber-related variables, clinical phenotype, physician-reported clinical severity, and baseline symptom burden, allowing an integrated assessment of formula use.

Several limitations should be acknowledged. First, this was an observational real-world study, and formula allocation was not randomized. Therefore, confounding by indication remains a major limitation. Pediatricians may have selected HRF or eHF based on clinical phenotype, perceived allergic risk, prior formula refusal, palatability, family preference, socioeconomic factors, availability, previous response, or perceived severity. Most of these variables were not measured, and residual confounding may persist despite multivariable adjustment and sensitivity analyses. Second, the CMPA diagnosis was based on routine clinical practice rather than a standardized challenge-confirmed definition in all cases. The study did not mandate oral food challenge, standardized elimination–reintroduction procedures, or centralized diagnostic adjudication. Consequently, misclassification is possible, including overdiagnosis of CMPA in infants with functional gastrointestinal disorders or under-recognition of atypical presentations. Third, CMPA phenotype and physician-reported clinical severity were not centrally adjudicated, and severity was not based on a validated CMPA severity instrument. Fourth, CoMiSS is not a diagnostic test for CMPA, and proposed thresholds have varied over time and across populations. Fifth, clinical evolution was assessed only at 7 days, limiting conclusions regarding sustained response, growth, long-term safety, nutritional adequacy, and tolerance acquisition. A 7-day assessment may also underestimate treatment response, particularly in non-IgE-mediated CMPA, in which gastrointestinal manifestations may require 2–4 weeks or longer to improve. Sixth, the complete-case analytical approach may have introduced selection bias if records with missing CoMiSS or key analytical variables differed systematically from included records. Finally, although adjusted analyses were performed, the available covariates could not capture all clinically relevant determinants of formula choice, and the comparative findings should therefore be considered exploratory and hypothesis-generating rather than definitive evidence of superiority, equivalence, or non-inferiority.

## 5. Conclusions

This real-world analysis from the ETAPA project suggests that HRF is used in routine pediatric practice as an additional nutritional option for infants with CMPA and is associated with clinically relevant short-term symptom improvement. HRF recommendation was slightly more likely in older infants and less likely in IgE-mediated CMPA, although the prescribing model had very low explanatory capacity and should be interpreted as exploratory. In the retrospective cohort, both HRF and eHF were associated with marked CoMiSS improvement after 7 days; however, several secondary outcomes suggested a faster or greater early symptomatic response with eHF. These differences should be interpreted cautiously because of the observational design, potential confounding by indication, baseline imbalances, and the absence of a superiority, equivalence, or non-inferiority framework. Overall, the findings support the feasibility and apparent short-term clinical usefulness of HRF in routine practice but should not be interpreted as evidence of equivalence or non-inferiority to eHF. Larger comparative studies using standardized diagnostic confirmation and longer follow-up are needed to assess sustained clinical response, growth, nutritional safety, and tolerance acquisition.

## Figures and Tables

**Table 1:** Description of the overall sample and the two analytical cohorts.

Variable	Overall	Prospective	Retrospective
*N*	1505	1094	411
Formula: eHF, *n* (%)	1085 (72.1)	880 (80.4)	205 (49.9)
Formula: HRF, *n* (%)	420 (27.9)	214 (19.6)	206 (50.1)
Male infant, *n* (%)	806 (55.4)	558 (53.3)	248 (60.8)
Female infant, *n* (%)	649 (44.6)	489 (46.7)	160 (39.2)
Infant age, months, median [IQR]	5.0 [3.0–8.0]	5.0 [3.0–8.0]	6.0 [3.0–9.0]
Age at diagnosis, months, median [IQR]	2.0 [1.0–4.0]	2.0 [2.0–4.0]	2.0 [1.0–4.0]
Diagnosis: non-IgE-mediated CMPA, *n* (%)	1037 (70.4)	748 (70.1)	289 (71.4)
Diagnosis: IgE-mediated CMPA, *n* (%)	435 (29.6)	319 (29.9)	116 (28.6)
Severity: mild, *n* (%)	544 (36.8)	414 (38.5)	130 (32.0)
Severity: moderate, *n* (%)	800 (54.1)	570 (53.1)	230 (56.7)
Severity: severe, *n* (%)	122 (8.2)	82 (7.6)	40 (9.9)
Severity: very severe, *n* (%)	14 (0.9)	8 (0.7)	6 (1.5)
Baseline CoMiSS, median [IQR]	10.0 [7.0–13.0]	9.0 [7.0–13.0]	10.0 [7.0–13.0]
Baseline CoMiSS ≥12, *n* (%)	526 (35.0)	375 (34.3)	151 (36.7)

Values are *n* (%) unless otherwise indicated; continuous variables are shown as median [IQR]. Percentages were calculated using available data for each variable. eHF, extensively hydrolyzed formula; HRF, hydrolyzed rice formula; CoMiSS, Cow’s Milk-related Symptom Score; IQR, interquartile range.

**Table 2:** Characteristics of the prospective recommendation cohort according to the formula recommended.

Variable	eHF	HRF	*p*-Value
*N*	880	214	
Infant age, months, median [IQR]	5.0 [3.0–7.0]	6.0 [3.0–8.5]	0.010
Age at diagnosis, months, median [IQR]	2.0 [1.0–4.0]	3.0 [2.0–5.0]	0.034
Infant sex: male	457 (54.0)	101 (50.2)	0.376
Infant sex: female	389 (46.0)	100 (49.8)	
CMPA phenotype: non-IgE-mediated	590 (68.8)	158 (75.2)	0.084
CMPA phenotype: IgE-mediated	267 (31.2)	52 (24.8)	
Severity: mild	338 (39.0)	76 (36.5)	0.666
Severity: moderate	453 (52.3)	117 (56.2)	
Severity: severe	69 (8.0)	13 (6.2)	
Severity: very severe	6 (0.7)	2 (1.0)	
Physician-reported ordinal severity score, median [IQR]	2.0 [1.0–2.0]	2.0 [1.0–2.0]	0.720
Baseline CoMiSS, median [IQR]	9.0 [6.0–12.2]	9.5 [7.0–13.0]	0.299
Baseline CoMiSS ≥12, *n* (%)	296 (33.6)	79 (36.9)	0.409
Prescriber age, years, median [IQR]	49.0 [39.0–57.0]	49.0 [38.8–56.2]	0.942
Prescriber sex: female	537 (61.9)	132 (62.9)	0.867
Prescriber sex: male	330 (38.1)	78 (37.1)	
Work setting: public	518 (60.0)	128 (61.2)	0.924
Work setting: private	175 (20.3)	42 (20.1)	
Work setting: both	171 (19.8)	39 (18.7)	
Facility type: primary care	426 (49.9)	107 (51.7)	0.984
Facility type: public hospital	167 (19.6)	41 (19.8)	
Facility type: private setting	180 (21.1)	40 (19.3)	
Facility type: mixed/other	64 (7.5)	15 (7.2)	
Facility type: other	16 (1.9)	4 (1.9)	
Professional profile: non-specialist	651 (75.8)	160 (76.6)	0.886
Professional profile: G/PA specialist	208 (24.2)	49 (23.4)	
Specialty: general pediatrics	601 (70.0)	148 (70.8)	0.965
Specialty: pediatric gastroenterology	172 (20.0)	39 (18.7)	
Specialty: pediatric allergology	36 (4.2)	10 (4.8)	
Specialty: family doctor working as a pediatrician	25 (2.9)	5 (2.4)	
Specialty: other	25 (2.9)	7 (3.3)	

Values are *n* (%) unless otherwise indicated; eHF, extensively hydrolyzed formula; HRF, hydrolyzed rice formula; CMPA, cow’s milk protein allergy; G/PA, pediatric gastroenterologist or pediatric allergologist; CoMiSS, Cow’s Milk-related Symptom Score; IQR, interquartile range.

**Table 3:** Multivariable logistic regression model for HRF recommendation versus eHF in the prospective cohort.

Variable	Univariate OR (95% CI)	*p*	Multivariate OR (95% CI)	*p*
Infant age, months	1.031 (1.000–1.063)	0.048	1.034 (1.002–1.067)	0.036
IgE-mediated CMPA	0.740 (0.516–1.063)	0.103	0.673 (0.459–0.988)	0.046
Physician-reported ordinal severity score	1.029 (0.800–1.323)	0.824	1.009 (0.765–1.332)	0.949
Baseline CoMiSS ≥12	1.131 (0.809–1.581)	0.471	1.242 (0.859–1.795)	0.250
Prescriber age, years	1.001 (0.987–1.016)	0.871	1.003 (0.988–1.019)	0.700
G/PA specialist prescriber	0.956 (0.658–1.388)	0.813	1.009 (0.597–1.707)	0.973

Hosmer–Lemeshow test: *p* = 0.012; Cox–Snell R^2^: 0.010; Nagelkerke R^2^: 0.016. Using the standard 0.50 classification threshold, the model correctly classified 80.7% of cases; sensitivity: 0.0%; specificity: 100.0%; positive predictive value (PPV): not estimable because no HRF cases were predicted; negative predictive value (NPV): 80.7%. The model included 960 complete cases, including 185 HRF recommendations. This table shows the univariate results of the variables finally included in the multivariate model. In this exploratory model, older infant age showed a small association with higher odds of HRF recommendation, whereas IgE-mediated CMPA showed an association with lower odds of HRF recommendation. However, the model showed poor calibration and very low explanatory capacity; therefore, these findings should not be interpreted as a predictive model of formula selection.

**Table 4:** Baseline characteristics of the retrospective treated cohort according to the formula received.

Variable	eHF	HRF	*p*-Value
*N*	205	206	
Infant age, months, median [IQR]	6.0 [3.0–9.25]	6.0 [3.0–9.0]	0.264
Age at diagnosis, months, median [IQR]	2.0 [1.0–3.0]	2.0 [1.0–4.0]	0.022
Infant sex: male	144 (70.6)	104 (51.0)	<0.001
Infant sex: female	60 (29.4)	100 (49.0)	
CMPA phenotype: non-IgE-mediated	150 (74.3)	139 (68.5)	0.239
CMPA phenotype: IgE-mediated	52 (25.7)	64 (31.5)	
Severity: mild	61 (30.0)	69 (34.0)	0.104
Severity: moderate	124 (61.1)	106 (52.2)	
Severity: severe	14 (6.9)	26 (12.8)	
Severity: very severe	4 (2.0)	2 (1.0)	
Physician-reported ordinal severity score, mean (SD)	1.8 (0.6)	1.8 (0.7)	1.000
Baseline CoMiSS, median (IQR)	10.0 [7.0–13.0]	10.0 [7.0–13.0]	0.127
Baseline CoMiSS ≥12, *n* (%)	66 (32.2)	85 (41.3)	0.071
Prescriber age, years, mean (SD)	47.8 (11.3)	48.0 (11.4)	0.815
Prescriber sex: female	119 (58.9)	123 (60.6)	0.808
Prescriber sex: male	83 (41.1)	80 (39.4)	
Work setting: public	116 (58.0)	122 (60.4)	0.797
Work setting: private	40 (20.0)	41 (20.3)	
Work setting: both	44 (22.0)	39 (19.3)	
Facility type: primary care	99 (49.7)	96 (48.5)	0.987
Facility type: public hospital	38 (19.1)	37 (18.7)	
Facility type: private setting	41 (20.6)	44 (22.2)	
Facility type: mixed/other	18 (9.0)	17 (8.6)	
Facility type: other	3 (1.5)	4 (2.0)	
Professional profile: non-specialist	149 (74.9)	153 (76.5)	0.793
Professional profile: G/PA specialist	50 (25.1)	47 (23.5)	
Specialty: general pediatrics	136 (68.3)	142 (71.0)	0.971
Specialty: pediatric gastroenterology	40 (20.1)	39 (19.5)	
Specialty: pediatric allergology	10 (5.0)	8 (4.0)	
Specialty: family doctor working as a pediatrician	6 (3.0)	5 (2.5)	
Specialty: other	7 (3.5)	6 (3.0)	

Values are *n* (%) unless otherwise indicated; eHF, extensively hydrolyzed formula; HRF, hydrolyzed rice formula; CMPA, cow’s milk protein allergy; G/PA, pediatric gastroenterologist or pediatric allergologist; CoMiSS, Cow’s Milk-related Symptom Score; IQR, interquartile range; SD: standard deviation.

**Table 5:** Clinical evolution from day 0 to day 7 in the retrospective treated cohort.

Variable	eHF	HRF	*p*-Value
*N*	205	206	
CoMiSS day 0, median [IQR]	10.0 [7.0–13.0]	10.0 [7.0–13.0]	0.127
CoMiSS day 7, median [IQR]	2.0 [1.0–4.0]	4.0 [1.0–5.8]	<0.001
Absolute CoMiSS reduction, median [IQR]	7.0 [4.0–10.0]	6.5 [4.0–10.0]	0.661
Percentage CoMiSS reduction, median [IQR]	76.5 [57.1–90.0]	69.2 [50.0–83.3]	0.003
CoMiSS reduction ≥50%, *n* (%)	170 (83.3)	154 (75.1)	0.051
CoMiSS day 7 <6, *n* (%)	172 (83.9)	154 (74.8)	0.028
CoMiSS day 7 ≥12, *n* (%)	4 (2.0)	3 (1.5)	0.724
Crying day 0, median [IQR]	3.0 [2.0–5.0]	3.0 [2.0–5.0]	0.389
Crying day 7, median [IQR]	1.0 [0.0–1.0]	1.0 [0.0–2.0]	0.477
Crying reduction, median [IQR]	2.0 [1.0–3.0]	2.0 [1.0–3.0]	0.093
Regurgitation day 0, median [IQR]	2.0 [1.0–4.0]	1.0 [0.0–4.0]	0.109
Regurgitation day 7, median [IQR]	0.0 [0.0–1.0]	0.0 [0.0–1.0]	0.017
Regurgitation reduction, median [IQR]	1.0 [0.0–3.0]	1.0 [0.0–3.0]	0.003
Stool score day 0, median [IQR]	4.0 [2.0–4.0]	4.0 [2.0–4.0]	0.287
Stool score day 7, median [IQR]	0.0 [0.0–2.0]	0.0 [0.0–2.0]	0.115
Stool score reduction, median [IQR]	2.0 [0.0–4.0]	2.0 [0.0–4.0]	0.946
Eczema head/neck/trunk day 0, median [IQR]	0.0 [0.0–1.0]	0.0 [0.0–1.0]	0.036
Eczema head/neck/trunk day 7, median [IQR]	0.0 [0.0–0.0]	0.0 [0.0–1.0]	0.032
Eczema head/neck/trunk reduction, median [IQR]	0.0 [0.0–0.0]	0.0 [0.0–1.0]	0.103
Eczema limbs day 0, median [IQR]	0.0 [0.0–0.0]	0.0 [0.0–1.0]	0.003
Eczema limbs day 7, median [IQR]	0.0 [0.0–0.0]	0.0 [0.0–0.0]	0.003
Eczema limbs reduction, median [IQR]	0.0 [0.0–0.0]	0.0 [0.0–1.0]	0.052
Urticaria day 0, median [IQR]	0.0 [0.0–0.0]	0.0 [0.0–0.0]	0.073
Urticaria day 7, median [IQR]	0.0 [0.0–0.0]	0.0 [0.0–0.0]	0.996
Urticaria reduction, median [IQR]	0.0 [0.0–0.0]	0.0 [0.0–0.0]	0.084
Respiratory symptoms day 0, median [IQR]	0.0 [0.0–0.0]	0.0 [0.0–0.0]	0.206
Respiratory symptoms day 7, median [IQR]	0.0 [0.0–0.0]	0.0 [0.0–0.0]	0.657
Respiratory symptoms reduction, median [IQR]	0.0 [0.0–0.0]	0.0 [0.0–0.0]	0.080

Positive reduction values indicate symptom improvement. Continuous variables are shown as median [IQR]. eHF, extensively hydrolyzed formula; HRF, hydrolyzed rice formula; CoMiSS, Cow’s Milk-related Symptom Score; IQR, interquartile range. Some comparisons showed identical medians but statistically significant *p*-values because the Mann–Whitney U test compares the overall rank distribution between groups rather than medians alone.

**Table 6:** Days to improvement, global clinical evolution, satisfaction, and phenotype-stratified outcomes in the retrospective cohort.

Variable	eHF	HRF	*p*-Value
Days to observe improvement	5.0 [3.0–7.0]	7.0 [4.0–10.0]	0.015
Global clinical evolution (1–4)	3.0 [3.0–4.0]	3.0 [3.0–4.0]	0.318
Product satisfaction (1–4)	4.0 [3.0–4.0]	4.0 [3.0–4.0]	0.158
Non-IgE-mediated subgroup			
Days to observe improvement	7.0 [4.0–9.5]	7.0 [5.0–12.0]	0.020
Global clinical evolution (1–4)	3.0 [3.0–4.0]	3.0 [3.0–4.0]	0.342
Product satisfaction (1–4)	4.0 [3.0–4.0]	4.0 [3.0–4.0]	0.191
Absolute CoMiSS reduction	7.0 [4.0–9.0]	6.0 [2.5–9.0]	0.044
IgE-mediated subgroup			
Days to observe improvement	3.0 [2.0–7.0]	4.0 [2.0–7.0]	0.165
Global clinical evolution (1–4)	4.0 [3.0–4.0]	4.0 [3.0–4.0]	0.543
Product satisfaction (1–4)	4.0 [4.0–4.0]	4.0 [3.0–4.0]	0.350
Absolute CoMiSS reduction	8.0 [5.0–11.0]	9.0 [6.0–12.0]	0.247

Scores for global clinical evolution and satisfaction range from 1 to 4, with higher values indicating better evolution or satisfaction. Continuous variables are shown as median [IQR]. eHF, extensively hydrolyzed formula; HRF, hydrolyzed rice formula; CoMiSS, Cow’s Milk-related Symptom Score; IQR, interquartile range.

## Data Availability

The original contributions presented in this study are included in the article/Supplementary Material. Further inquiries can be di-rected to the corresponding author.

## Supplementary Materials

The following supporting information can be downloaded at: https://www.mdpi.com/article/10.3390/nu18132137/s1, Supplementary Table S1. Nutritional composition of the formulas evaluated in the ETAPA project. Supplementary Table S2. Sociodemographic characteristics of the participating pediatricians. Supplementary Table S3. Summary of the main differences observed between eHF and HRF in the ETAPA analysis. Supplementary Table S4. Baseline covariate balance before and after propensity score weighting in the retrospective treated cohort.

## Abbreviations

AAF, amino acid formula; CI, confidence interval; CMP, cow’s milk protein; CMPA, cow’s milk protein allergy; CoMiSS, Cow’s Milk-related Symptom Score; DRACMA, Diagnosis and Rationale for Action against Cow’s Milk Allergy; eHF, extensively hydrolyzed formula; ESPGHAN, European Society for Paediatric Gastroenterology, Hepatology and Nutrition; G/PA, pediatric gastroenterologist or pediatric allergologist; HRF, hydrolyzed rice formula; IgE, immunoglobulin E; IQR, interquartile range; OFC, oral food challenge; OR, odds ratio; VIF, variance inflation factor; y SMD, standardized mean difference; PPV, positive predictive value; NPV, negative predictive value.
