# Supplementary material for: Real-World Use, Prescribing Patterns, and Short-Term Clinical Evolution of Extensively Hydrolyzed and Hydrolyzed Rice Formulas in Infants with Cow’s Milk Protein Allergy: An Analysis from the ETAPA Project
Source: Nutrients. 2026 Jul 2;18(13):2137. doi: 10.3390/nu18132137 (PMC13363393; doi:10.3390/nu18132137)
Supplement: Supplementary file 1 [file nutrients-18-02137-s001.zip › nutrients-4376582-supplementary.pdf]

Supplementary Table S1. Nutritional composition of the formulas evaluated in the ETAPA project

Section	Component / parameter	Unit	HRF evaluated in the study	eHF evaluated in the study
Formula and preparation				
	Formula type		Hydrolyzed rice protein formula (HRF)	Extensively hydrolyzed formula (eHF)
	Protein hydrolysate source		Rice protein hydrolysate	Cow's milk protein hydrolysate
Energy and macronutrients				
	Energy	kcal	67.7	69
	Energy	kJ	283	289
	Total protein	g	1.62	1.75
	Total fat	g	3.44	3.50
	MCTs	g	0.95	0.72
	Linoleic acid (C18:2, omega-6)	mg	382	428
	Alpha-linolenic acid (C18:3, omega-3)	mg	39	52
	Arachidonic acid (AA)	mg	NA	15.68
	Docosahexaenoic acid (DHA)	mg	16.2	15.68
	Gamma-Linolenic acid (GLA)	mg	NA	9.8
	Total carbohydrates	g	7.36	7.42
	Maltodextrin	g	5.74	7.42
	Corn starch	g	1.62	NA
	Total fiber	g	0.41	0.42
	Fructooligosaccharides	g	0.20	0.21
	Inulin	g	0.20	0.21
	Choline	mg	20.25	18.2
	Taurine	mg	4.73	4.48
	Inositol	mg	3.4	3.50
	L-carnitine	mg	0.95	1.05
Minerals				
	Sodium	mg	30.4	25.9
	Potassium	mg	67.5	70.7
	Chloride	mg	56.7	46.9
	Calcium	mg	74.3	86.8
	Phosphorus	mg	48.6	56.0
	Iron	mg	0.7	0.6
	Magnesium	mg	6.1	6.2
	Zinc	mg	0.7	0.7
	Copper	µg	50	49.7
	Iodine	µg	13.5	14.0
	Manganese	µg	20.3	14.0
	Selenium	µg	2.7	2.7
	Chromium	µg	2.7	2.8
	Molybdenum	µg	4.1	4.2
	Calcium/phosphorus ratio		1.5	1.9

Section	Component / parameter	Unit	HRF evaluated in the study	eHF evaluated in the study
		Vitamins		
	Vitamin A	µg/IU	59.4/198	61.6/205.3
	Vitamin D	µg/IU	1.5/59.6	1.5/61.6
	Vitamin E	mg/IU	1/1.4	1/1.4
	Vitamin K	µg	5.4	5.6
	Vitamin B1 (thiamine)	µg	60.8	107.8
	Vitamin B2 (riboflavin)	µg	155.3	161.0
	Vitamin B6 (pyridoxine)	µg	103.4	107.8
	Vitamin B12 (cobalamin)	µg	0.1	0.1
	Vitamin C	mg	11.5	11.9
	Folic acid	µg	12.2	12.6
	Pantothenic acid	mg	0.4	0.4
	Niacin	mg	0.7	0.7
	Biotin	µg	1.2	1.3
		Nucleotides		
	Cytidine-5'-monophosphate	mg	0.9	1
	Uridine-5'-monophosphate	mg	0.7	0.7
	Adenosine-5'-monophosphate	mg	0.3	0.3
	Guanosine-5'-monophosphate	mg	0.2	0.2
	Inosine-5'-monophosphate	mg	0.2	0.2
		Other parameters		
	Total probiotics	CFU	2.55E+09 per 100 g powder	2.55E+09 per 100 g powder
	B. infantis CECT720	CFU	3.00E+08 per 100 g powder	3.00E+08 per 100 g powder
	L. rhamnosus HN001	CFU	2.25E+09 per 100 g powder	2.25E+09 per 100 g powder
	Osmolarity	mOsm/L	111	240
		Fatty acid profile, per 100 g of fatty acids		
	Caprylic acid (C8:0)	%	16.2	12.2
	Capric acid (C10:0)	%	11.1	8.3
	Lauric acid (C12:0)	%	8.2	8.8
	Myristic acid (C14:0)	%	3.3	3.5
	Palmitic acid (C16:0)	%	4.1	4.4
	Stearic acid (C18:0)	%	2.0	2.2
	Oleic acid (C18:1)	%	33.2	35.5
	Linoleic acid (C18:2)	%	11.1	12.2
	Alpha-linolenic acid (C18:3)	%	1.1	1.5
	Arachidonic acid (AA)	%	Not reported	0.448
	Docosahexaenoic acid (DHA)	%	0.5	0.448
	Gamma-linoleic (GLA)	%	Not reported	0.280
	Other fatty acids	%	8.3	10.2
	Saturated fatty acids	%	45.3	39.4
	Monounsaturated fatty acids	%	33.2	35.5
	Polyunsaturated fatty acids	%	21.5	25.1
	MCTs	%	27.6	20.6

Abbreviations: AA, arachidonic acid; CFU, colony-forming units; DHA, docosahexaenoic acid; eHF, extensively hydrolyzed formula; HRF, hydrolyzed rice formula; MCTs, medium-chain triglycerides.

Values were transcribed from the manufacturer's product-analysis datasheets provided for the HRF and eHF formulas. "NA" indicates that the compound is not present in the formula. Values are shown per 100 mL of reconstituted formula unless otherwise specified. The HRF datasheet labels most per-100 mL columns as 13.5% reconstitution, whereas the eHF datasheet specifies 14%. Fatty acid profiles are expressed per 100 g of fatty acids, as reported in the product-analysis datasheets. "NA" indicates that the compound is not present in the formula.

Supplementary Table S2. Sociodemographic characteristics of the participating pediatricians

	**Pediatricians (N=269)**
**Age (years old), mean (SD)***	48.3 (11.0)
**Sex, n (%)*****	
Female	165 (62.3)
Male	100 (37.7)
**Work setting, n (%)***	
Public	158 (60.1)
Private	52 (19.8)
Both	53 (20.2)
**Location of the workplace, n (%)****	
Urban	230 (87.8)
Semi-urban	28 (10.7)
Rural	4 (1.5)
**Type of facility**	
Primary Care	149 (55.4)
Public Hospital	54 (20.1)
Private Practice	48 (17.8)
Private Hospital	18 (6.7)
Other	12 (4.5)
**Specialty****	
General Pediatrics	187 (71.4)
Pediatric Gastroenterology	50 (19.1)
Pediatric Allergology	11 (4.2)
Family Doctor working as pediatrician	7 (2.7)
Others	7 (2.7)

Total number of valid responses: *N=263; **N=262; ***N=265. SD: standard deviation

Supplementary Table S3. Summary of the main differences observed between eHF and HRF in the ETAPA analysis.

Analytical component	Outcome/domain	eHF	HRF	Interpretation
Prospective recommendation cohort	HRF recommendation	880/1094 eHF recommendations (80.4%)	214/1094 HRF recommendations (19.6%)	HRF represented approximately one fifth of real-world recommendations.
Prospective recommendation cohort	Infant age	5.0 [3.0-7.0] months	6.0 [3.0-8.5] months	Infants recommended HRF were slightly older (p=0.010).
Prospective recommendation cohort	IgE-mediated CMPA	31.2%	24.8%	IgE-mediated CMPA was less frequent among HRF recommendations; adjusted OR for HRF recommendation 0.673 (0.459-0.988)
Prospective recommendation cohort	Baseline CoMiSS	9.0 [6.0-12.2]	9.5 [7.0-13.0]	No significant difference in baseline symptom burden (p=0.299).
Retrospective treated cohort	Baseline CoMiSS	10.0 [7.0-13.0]	10.0 [7.0-13.0]	Similar baseline symptom burden (p=0.127).
Retrospective treated cohort	CoMiSS day 7	2.0 [1.0-4.0]	4.0 [1.0-5.8]	Lower day 7 CoMiSS with eHF (p<0.001).
Retrospective treated cohort	Absolute CoMiSS reduction	7.0 [4.0-10.0]	6.5 [4.0-10.0]	Similar absolute reduction (p=0.661); adjusted beta for HRF -0.513 points (95% CI -1.108 to 0.082).
Retrospective treated cohort	Percentage CoMiSS reduction	76.5 [57.1-90.0]%	69.2 [50.0-83.3]%	Greater percentage reduction with eHF (p=0.003).
Retrospective treated cohort	CoMiSS reduction ≥50%	83.3%	75.1%	Borderline unadjusted difference (p=0.051); adjusted OR for HRF 0.576 (95% CI 0.335-0.989).

Analytical component	Outcome/domain	eHF	HRF	Interpretation
Retrospective treated cohort	CoMiSS day 7 <6	83.9%	74.8%	More frequent symptomatic normalization with eHF (p=0.028).
Retrospective treated cohort	CoMiSS day 7 ≥12	2.0%	1.5%	Persistence of high CoMiSS was uncommon in both groups (p=0.724).
Retrospective treated cohort	Days to observed improvement	5.0 [3.0-7.0]	7.0 [4.0-10.0]	Earlier observed improvement with eHF (p=0.015).
Retrospective treated cohort	Global clinical evolution	3.0 [3.0-4.0]	3.0 [3.0-4.0]	High and similar global evolution scores (p=0.318).
Retrospective treated cohort	Product satisfaction	4.0 [3.0-4.0]	4.0 [3.0-4.0]	High and similar satisfaction scores (p=0.158).

Supplementary Table S4. Baseline covariate balance before and after propensity score weighting in the retrospective treated cohort

Variable	SMD before weighting	SMD after weighting
Infant age (months)	0.094	0.025
Infant sex	0.343	0.009
Age at diagnosis (months)	0.223	0.016
CMPA phenotype	0.163	0.003
Physician-reported ordinal severity score	0.027	0.009
Baseline CoMiSS	0.181	0.007
Prescriber age (years)	0.022	0.010
G/PA specialist profile	0.010	0.010
Work setting	0.031	0.016
Type of facility	0.095	0.009

Propensity score-weighted sensitivity analysis sample: 357 complete cases from the retrospective treated cohort (eHF, n = 179; HRF, n = 178). Stabilized inverse probability of treatment weighting was used.

Notes: Values are absolute standardized mean differences. For categorical variables with more than two categories, the value shown corresponds to the maximum absolute category-specific standardized mean difference. Values <0.10 were considered indicative of adequate covariate balance. The propensity score model included infant age, infant sex, age at diagnosis, CMPA phenotype, physician-reported ordinal severity score, baseline CoMiSS, prescriber age, G/PA specialist profile, work setting, and type of facility.

Abbreviations: CMPA, cow's milk protein allergy; CoMiSS, Cow's Milk-related Symptom Score; eHF, extensively hydrolyzed formula; G/PA, pediatric gastroenterologist or pediatric allergologist; HRF, hydrolyzed rice formula; SMD, standardized mean difference.

≥
